# Spatial disparity dynamics of ecosystem service values and GDP in Shaanxi Province, China in the last 30 years

**DOI:** 10.1371/journal.pone.0174562

**Published:** 2017-03-30

**Authors:** Tianhong Li, Yao Ding

**Affiliations:** 1 College of Environmental Sciences and Engineering, Peking University, Beijing, China; 2 Key Laboratory of Water and Sediment Sciences, Ministry of Education, Beijing, China; 3 Shenzhen Graduate School of Peking University, Shenzhen, Guangdong, China; University of Waikato, NEW ZEALAND

## Abstract

The regional policy in China is shifting from solely gross domestic product (GDP) orientation to development that is more balanced between economic growth and ecological protection, as well as achieving equality among regions. Using land use maps and the adjusted value coefficients to assess ecosystem service values (ESV) for the 1980s, 1995, 2000, and 2010, we estimated the ESV in Shaanxi Province for different years, and characterized the spatial and temporal distribution of ESV and GDP. The results demonstrated that the total value of ecosystem services in Shaanxi Province increased from 208.95 billion Yuan in the 1980s to 309.76 billion Yuan in 2010. Variation Coefficient (*C*_*v*_) and Theil index (T) were used to reflect the disparities of GDP or ESV within the study area. The values of *Cv* in descending order are GDP, ESV per capita, ESV, and GDP per capita. The Theil indexes of GDP were much greater than the ones of ESV. Variations of *Cv* and T showed that disparity in GDP kept increasing from the 1980s to 2000, then decreased; while no significant change in regional disparity of ESV were detected in parallel. The cities with higher GDP usually contributed little to ESV, and vice versa. The variation in GDP and ESV, in terms of the prefectural totals and per capita values, increased from the 1980s to 2010. This study provides an accessible way for local decision makers to evaluate the regional balance between economic growth and ecosystem services.

## Introduction

Ecosystem services can be defined as the goods and services provided by ecosystems, which contribute to human welfare, both directly and indirectly [1], or the conditions and processes through which natural ecosystems and the species that comprise them to sustain and fulfill human life [2]. The concept of ecosystem services has been increasingly used to highlight, measure, and value the degree of interdependence between humans and the rest of nature [3, 4]. Valuation of ecosystem services is useful to seek choices to manage natural and human-made capital more effectively and sustainably [5]. It has become a subject of increased interest in the field of ecology, environmental economics and sustainable development [6].

When ecological services is valued over large spatial and temporal scales, the method for aggregating values are often used [5]. Kubiszewski et al. generalized this kind of method into four levels including basic value transfer, expert modified value transfer, statistical value transfer, and spatially explicit functional modelling [7]. Costanza et al. proposed the basic benefit transfer approach, which assumes a constant unit value per hectare of ecosystem type and multiples that value by the area of each type to arrive at aggregate totals [1, 8]. It has been widely used due to the simplicity in calculation [9–14]. Xie et al. adapted the basic value transfer method based on a survey on 700 Chinese ecologists to reflect the actual situations of China [15, 16]. Other researchers applied this expert modified value transfer method to calculate ESV in various regions of China [17–24].

Land use data can be relatively easily retrieved using cost-effective remote sensing images. The land use type can act as a proxy for ecosystem services by matching the land use types to equivalent biomes [25], which facilitates the valuation of ecosystem services for various spatial scales. In addition to global scale studies [26, 27], many other studies [17–23, 28–30] have been conducted at regional or even local scales. Most studies have focused on ESV variations in response to land use changes [19–21], which provide useful information for sustainable land management [31].

More recent works [22, 32, 33] tried to address both ecological service and economic performance in their study areas. Follow this strand of research, this paper presents the changing spatial disparities in both ESV and local economic development in Shaanxi, China since 1980s up to 2010.

As an underdeveloped region with fragile environment, Shaanxi has long suffered from severe soil and water erosion. It is the key area of a national eco-environmental improvement project called Grain for Green (GfG, also known as the project to return farmland to woodland and grassland) [34] or Sloping Land Conversion Program (SLCP) [35] that is regarded as the largest land-use transition program in the world in recent decades [36, 37]. In the past three decades, dramatic changes in land use have taken place in the province. Previous studies [18, 34, 38, 39] on land use and ecological services issues focused on the northern part of the province and on the influence of GfG project. In recent years, the regional policy in China is shifting from solely GDP oriented toward development that balanced between economic growth and ecological protection, as well as regional equality. The value of ecological service should be considered in policy making in balancing regions with different natural endowment. Thus, it calls for the examination of the relation between spatial disparities of ESV and economic criteria, such as regional GDP.

With the expert modified value transfer method [15,16] for ESV estimating, and time series (1980s, 1995, 2000, and 2010) of land use maps and statistical datasets, the present study aims to reveal the temporal and spatial variations of the ESV and to compare spatial disparities of GDP and ESV between 10 prefecture-level cities of Shaanxi Province. The results will provide policy-makers with insights for balancing regional disparities of the ESV and economic development to achieve equitable development.

## Materials and methods

### Study site

**Fig 1:**
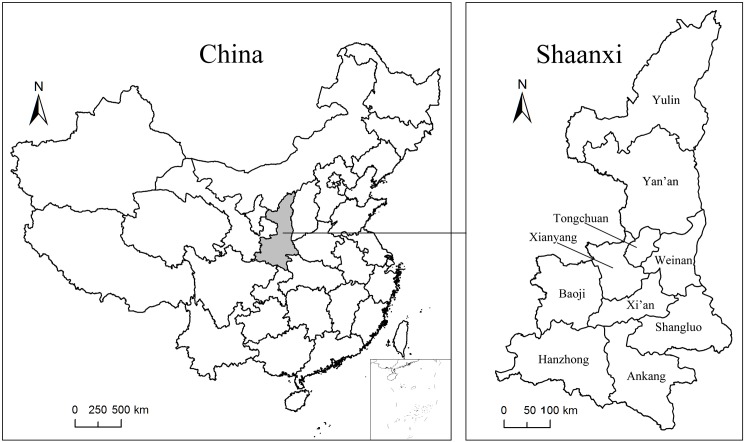
The location of the study area.

Shaanxi Province (31° 42 ‘~39° 35’ N, 105° 29 ‘~ 111° 15’ E) is located in the northwest of inland China, crossing the Yangtze River basin and the Yellow River basin. It covers approximately 205,600 km^2^, with a population of 37.35 million in 2010. With Shanxi and Henan in the east, Ningxia and Gansu in the west, Sichuan, Chongqing, and Hubei in the south and Inner Mongolia in the north, Shaanxi Province is a geographically important hub linking the Eastern and Central China to the Northwest and Southwest regions. Shaanxi province consists of ten prefectural cities. They are Yulin, Yan’an, Xi’an, Tongchuan, Baoji, Xianyang, Weinan, Hanzhong, Ankang and Shangluo covering 21.09%, 17.93%, 4.89%, 1.88%, 8.77%, 4.96%, 6.36%, 13.19%, 11.32% and 9.61% of the total area respectively. Yulin and Yan’an in the northern Shaanxi comprises the core part of the Loess Plateau. The Qinling and the Bashan mountains are mainly distribute in Hanzhong, Ankang and Shangluo in the south. Xi’an, Xianyang, Baoji, Tongchuan and Weinan are mainly occupied by Loess hilly areas, alluvial plain and valleys. Serious soil and water loss take place in the northern part. In 1999, Chinese government launched GfG project to mitigate and prevent soil erosion and other ecological degradation problems in Shaanxi Province [40]. The project stipulated that sloped cropland and cropland unsuitable for farming should be gradually converted into woodland and grassland [41]. In the past decades, the land use/cover has changed dramatically. The location of Shaanxi Province is shown in **Fig 1**.

### Data processing and demonstration

**Fig 2:**
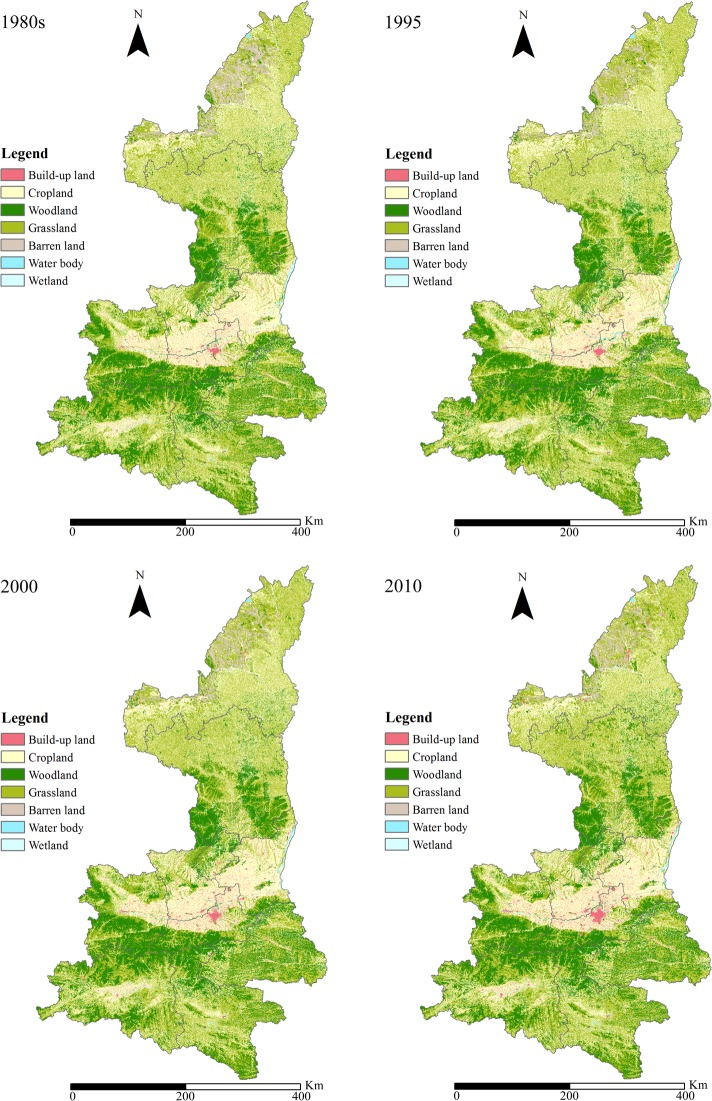
Land use maps of Shaanxi Province in the 1980s, 1995, 2000, and 2010.

Four land use datasets in the 1980s, 1995, 2000, and 2010 on the scale of 1:100, 000 were from Data Sharing Infrastructure of Earth System Science, China. These data sets were produced with standard interpretation procedures based on Landsat TM\ETM+ images [42]. The accuracy of the six classes of land use was above 94.3%, and the overall accuracy of the 25 subclasses was above 91.2%, which can meet the requirement of mapping accuracy on the 1:100, 000 scale and widely used in land use change studies in China [43]. The original 25 land use subclasses include paddy fields, dry land, forestland, high coverage grassland, canals, urban land, sandy land, and others. According to the actual circumstances of land use and the study area, these land use maps were georeferenced to the same projection and reclassified in ArcGIS 9.3 (ESRI, Redlands, CA) into 7 types, specifically, Woodland, Grassland, Cropland, Wetland, Water body, Barren land, and Build-up land **(Fig 2)**. The table **in S1 Table** shows how the original land use classes were reclassified to the 7 types used in this study.

The border of Shaanxi province and its 10 prefectural cities was derived from a digital version of the Chinese Administrative Zoning Map at a scale of 1:100,000, which was obtained from the Chinese Geographic Information Center. The social and economic data sets such as population and GDP for the ten cities and the whole province were obtained from Chinese and Shaanxi statistical yearbooks [44, 45]. To enhance the comparability of GDP, data of GDP in the 1980s, 1995, and 2000 is converted to 2010 benchmark.

### Ecosystem services value evaluation method

The ESV estimating method used in references [15–21] was adopted in this study. Considering the actual situation of the study area, ecosystem service values per unit area for different land use types were determined. Xie et al. extracted the equivalent-weighting factor of ecosystem services per hectare of terrestrial ecosystems in China in 2002 based on a questionnaire survey of 200 Chinese ecologists, and they adjusted these factors in 2007 based on extensive questionnaire survey of 500 Chinese ecologist [15, 16]. The adjusted equivalent weighting factors of ecosystem services per hectare of terrestrial ecosystems in China (**Table 1**) are used in this study.

**Table 1:** Equivalent weighting factors of ecosystem services per hectare of terrestrial ecosystems in China [16].

	Woodland	Grassland	Cropland	Wetland	Water body	Barren land	Build-up land
**Gas regulation**	4.32	1.50	0.72	2.41	0.51	0.06	0.06
**Climate regulation**	4.07	1.56	0.97	13.55	2.06	0.13	0.13
**Water supply**	4.09	1.52	0.77	13.44	18.77	0.07	0.07
**Soil formation and protection**	4.02	2.24	1.47	1.99	0.41	0.17	0.17
**Waste treatment**	1.72	1.32	1.39	14.40	14.85	0.26	0.26
**Biodiversity protection**	4.51	1.87	1.02	3.69	3.43	0.40	0.40
**Food**	0.33	0.43	1.00	0.36	0.53	0.02	0.02
**Raw material**	2.98	0.36	0.39	0.24	0.35	0.04	0.04
**Recreation and culture**	2.08	0.87	0.17	4.69	4.44	0.24	0.24
**Total**	28.12	11.67	7.90	54.77	45.35	1.39	1.39

One factor is equal to the economic value of average food production of cropland per hectare per year under natural conditions. The natural food production is proposed to be 1/7 of the actual food production [15, 21]. Thus the economic value of one factor is calculated by the food price (Yuan per hectare) in the market multiplying with the 1/7 of actual crop yield in a hectare.

In contrast to most studies of dynamic evaluation of ESVs [17, 19, 21, 23], which have focused on changes in the ESV in response to land use changes and have adopted the same values per hectare for ecosystem services for different years, the present study focuses on ESV disparities among regions and compares them with the disparity in the GDP. Therefore, different values of ecosystem services per hectare are used for different years. The unit prices for food production for farmland ecosystem services were 718.01 Yuan·ha^-1^·yr^-1^, 658.99 Yuan·ha^-1^·yr^-1^, 695.43 Yuan·ha^-1^·yr^-1^, and 1064.69 Yuan ·ha^-1^·yr^-1^ for the 1980s, 1995, 2000, and 2010, respectively. Wheat and corn were used as the main food crop species because they dominated the sowing area in the study area, and we combined the yield data, seeding area and price obtained from the statistical yearbook [44, 45]. To enhance the comparability of the data, all unit price data per year were converted to 2010 benchmark. Thereby, the unit price of the ecosystem services in Shaanxi province from the 1980s to 2010 can be acquired as shown in **Table 2**.

**Table 2:** Economic value per hectare of ecosystem services of Shaanxi from the 1980s to 2010 (Yuan·ha^-1^·yr^-1^).

	Woodland	Grassland	Cropland	Wetland	Water body	Barren land	Build-up land
**1980s**							
**Gas regulation**	3101.80	1077.02	516.97	1730.40	366.19	43.08	43.08
**Climate regulation**	2922.30	1120.10	696.47	9729.04	1479.10	93.34	93.34
**Water supply**	2936.66	1091.38	552.87	9650.05	13477.05	50.26	50.26
**Soil formation and protection**	2886.40	1608.34	1055.47	1428.84	294.38	122.06	122.06
**Waste treatment**	1234.98	947.77	998.03	10339.34	10662.45	186.68	186.68
**Biodiversity protection**	3238.23	1342.68	732.37	2649.46	2462.77	287.20	287.20
**Food**	236.94	308.74	718.01	258.48	380.55	14.36	14.36
**Raw material**	2139.67	258.48	280.02	172.32	251.30	28.72	28.72
**Recreation and culture**	1493.46	624.67	122.06	3367.47	3187.96	172.32	172.32
**Total**	20190.44	8379.18	5672.27	39325.4	32561.75	998.02	998.02
**1995**							
**Gas regulation**	2846.84	988.49	474.47	1588.17	336.08	39.54	39.54
**Climate regulation**	2682.09	1028.02	639.22	8929.31	1357.52	85.67	85.67
**Water supply**	2695.27	1001.66	507.42	8856.83	12369.24	46.13	46.13
**Soil formation and protection**	2649.14	1476.14	968.72	1311.39	270.19	112.03	112.03
**Waste treatment**	1133.46	869.87	916.00	9489.46	9786.00	171.34	171.34
**Biodiversity protection**	2972.04	1232.31	672.17	2431.67	2260.34	263.60	263.60
**Food**	217.47	283.37	658.99	237.24	349.26	13.18	13.18
**Raw material**	1963.79	237.24	257.01	158.16	230.65	26.36	26.36
**Recreation and culture**	1370.70	573.32	112.03	3090.66	2925.92	158.16	158.16
**Total**	18530.8	7690.42	5206.03	36092.89	29885.2	916.01	916.01
**2000**							
**Gas regulation**	3004.26	1043.15	500.71	1675.99	354.67	41.73	41.73
**Climate regulation**	2830.40	1084.87	674.57	9423.08	1432.59	90.41	90.41
**Water supply**	2844.31	1057.05	535.48	9346.58	13053.22	48.68	48.68
**Soil formation and protection**	2795.63	1557.76	1022.28	1383.91	285.13	118.22	118.22
**Waste treatment**	1196.14	917.97	966.65	10014.19	10327.14	180.81	180.81
**Biodiversity protection**	3136.39	1300.45	709.34	2566.14	2385.32	278.17	278.17
**Food**	229.49	299.03	695.43	250.35	368.58	13.91	13.91
**Raw material**	2072.38	250.35	271.22	166.90	243.40	27.82	27.82
**Recreation and culture**	1446.49	605.02	118.22	3261.57	3087.71	166.90	166.90
**Total**	19555.49	8115.65	5493.9	38088.71	31537.76	966.65	966.65
**2010**							
**Gas regulation**	4599.46	1597.04	766.58	2565.90	542.99	63.88	63.88
**Climate regulation**	4333.29	1660.92	1032.75	14426.55	2193.26	138.41	138.41
**Water supply**	4354.58	1618.33	819.81	14309.43	19984.23	74.53	74.53
**Soil formation and protection**	4280.05	2384.91	1565.09	2118.73	436.52	181.00	181.00
**Waste treatment**	1831.27	1405.39	1479.92	15331.54	15810.65	276.82	276.82
**Biodiversity protection**	4801.75	1990.97	1085.98	3928.71	3651.89	425.88	425.88
**Food**	351.35	457.82	1064.69	383.29	564.29	21.29	21.29
**Raw material**	3172.78	383.29	415.23	255.53	372.64	42.59	42.59
**Recreation and culture**	2214.56	926.28	181.00	4993.40	4727.22	255.53	255.53
**Total**	29939.09	12424.95	8411.05	58313.08	48283.69	1479.93	1479.93

Once the ecosystem service value of per unit area for each land use category has been determined, the service value for each land use type, and service function are given in **Eq (1)** and **Eq (2)**, and the total ESV can be aggregated with **Eq (3)** [13, 15, 19, 21].

$$ESV_{k}=\sum_{f}A_{k}\times VC_{kf}$$ (1)

$$ESV_{f}=\sum_{k}A_{k}\times VC_{kf}$$ (2)

$$ESV_{}=\sum_{k}\sum_{f}A_{k}\times VC_{kf}$$ (3)

*ESV*_*k*_, *ESV*_*f*_ and *ESV* refer to the ecosystem service value of land use category “*k*”, value of ecosystem service function type “*f*” and the total ecosystem service value respectively. *A*_*k*_ is the area (ha) for land use category “*k”* and *VC*_*kf*_ is the value coefficient (Yuan·ha^-1^·yr^-1^) for land use category “*k”* and ecosystem service function type “*f”*, *and VC*_*kf*_ is defined in **Table 2**.

### Regional disparity of the GDP and ESV

Two indices, Variation Coefficient (*C*_*v*_) and Theil index (T) are used to reflect the disparities of GDP or ESV within Shaanxi Province.

*C*_*v*_ is independent of the unit in which the measurement has been taken, so it is a dimensionless number, and is appropriate for comparison between data sets with different units or widely different means. In this paper, *C*_*v*_ is used to describe the dispersive or concentrative extent of GDP or ESV in Shaanxi Province. It is defined by **Eq (4)**.

$$C_{v}=\frac{1}{{\text{Y}}_{0}}\sqrt{\frac{\sum_{i=1}^{n}{\left(Y_{i}-{\text{Y}}_{0}\right)}^{2}}{n}}$$ (4)

Where *n* is number of cities, here n = 10; Y_0_ is the mean value of GDP or ESV of the province; Y_i_ is the value of GDP or ESV of the *i*th city. The greater the value of *Cv* of GDP or ESV is, the greater dispersive and concentrative extent the GDP or ESV of the study area has. Since *C*_*v*_ did not considered the area of the cities, the Theil index is also used in this study.

The Theil index was proposed by econometrician Henri Theil, and it is a statistic used to measure economic inequality [46, 47]. One of the advantages of the Theil index is that it is a weighted average of inequality within subgroups, plus inequality among those subgroups. Different to the original Theil index, which is to measure the inequality among populations, the present study uses the Theil index to measure the inequality among cities. Considering the huge difference in area between the cities in Shaanxi, Theil index is defined by **Eq (5)**

T=∑i=1nYiYlnYiYSiS (5)

Where *n* is number of cities; *Y* is the total value of the GDP or ESV of the province; *Y*_*i*_ is the value of the GDP or ESV of the *i*th city, *Si* is the area of the *i*th city, and *S* is the total area of the province. The value of this index ranges between 0 and 1, and the larger values indicate a larger disparity in the GDP or ESV in the study area.

## Results and discussion

### Land use dynamics

**Table 3** summarized the land use changes in percentages in Shaanxi Province from the 1980s until 2010. Cropland, Grassland, and Woodland were the three predominating land use types in the study area during the study period. The other four land use types comprised approximately 5% of the total area each year. It was also obvious that Build-up land continuously increased from the 1980s to 2010. This variation was highly influenced by urbanization process. The areal percentages of Woodland and Grassland increased from 60.32% to 61.38% from 2000 to 2010 while Cropland decreased from 34.97% to 33.18% during the same period, this variation was closely related to GfG project [33].

**Table 3:** Land use percentages in Shaanxi Province from the 1980s to 2010 (%).

Year	Woodland	Grassland	Cropland	Wetland	Water body	Barren land	Build-up land
**1980s**	22.43	37.49	34.90	0.37	0.53	2.93	1.34
**1995**	22.09	38.81	34.78	0.32	0.59	1.97	1.44
**2000**	22.53	37.79	34.97	0.36	0.50	2.31	1.53
**2010**	23.34	38.34	33.18	0.35	0.51	2.19	2.09

The dynamics of land use during the last 30-year period were calculated using the map algebra of the ArcGIS 9.3 software (ESRI, Redlands, CA). Through change detection analysis, the land use conversion matrix between two snapshots can be produced [48]. The conversion matrix of land use types of Shaanxi Province from the 1980s to 2010 is shown in **Table 4**; other conversion matrices from the 1980s to 1995, 1995 to 2000, and 2000 to 2010 are shown in **Tables 5**, **6** and **7,** respectively.

**Table 4:** The conversion matrix of land use types in Shaanxi Province from the 1980s to 2010 (km^2^).

Land use type in 1980s	Land use type in 2010						
	Woodland	Grassland	Cropland	Wetland	Water body	Barren land	Build-up land
**Woodland**	44824.20	711.49	499.68	4.99	8.65	25.85	80.11
**Grassland**	1514.79	72000.22	3231.39	28.34	69.59	119.35	154.08
**Cropland**	1550.64	4639.24	64049.99	45.82	104.18	38.43	1379.18
**Wetland**	3.60	42.99	111.81	456.24	129.38	0.82	6.59
**Water body**	8.46	61.87	112.70	174.66	718.62	4.52	6.12
**Barren land**	83.30	1428.74	108.35	3.16	12.94	4321.88	77.59
**Build-up land**	23.63	19.18	138.28	1.71	4.27	0.08	2576.10

**Table 5:** The conversion matrix of land use types in Shaanxi Province from the 1980s to 1995 (km^2^).

Land use type in 1980s	Land use type in 1995						
	Woodland	Grassland	Cropland	Wetland	Water body	Barren land	Build-up land
**Woodland**	44694.85	1154.03	242.49	6.96	3.36	17.45	38.57
**Grassland**	389.34	75016.11	1581.38	22.57	29.72	57.20	25.73
**Cropland**	302.81	1667.92	69135.73	45.53	107.26	12.85	537.35
**Wetland**	1.17	50.35	58.08	461.84	166.42	7.57	6.88
**Water body**	2.96	30.90	26.86	116.31	902.71	8.10	2.60
**Barren land**	45.50	1894.89	127.84	0.35	6.10	3958.48	3.39
**Build-up land**	11.12	29.49	380.02	0.72	2.89	0.06	2338.95

**Table 6:** The conversion matrix of land use types in Shaanxi Province from 1995 to 2000 (km^2^).

Land use type in 1995	Land use type in 2000						
	Woodland	Grassland	Cropland	Wetland	Water body	Barren land	Build-up land
**Woodland**	44873.41	255.87	290.20	1.08	2.12	9.57	15.50
**Grassland**	1152.01	76148.49	1755.04	31.04	16.80	700.08	40.22
**Cropland**	262.12	1196.32	69422.99	47.97	19.67	109.27	494.08
**Wetland**	6.49	19.18	58.84	514.25	54.48	0.00	1.04
**Water body**	3.24	20.94	117.22	142.40	925.85	5.70	3.12
**Barren land**	32.75	75.47	13.41	5.37	7.11	3927.52	0.06
**Build-up land**	27.77	19.02	300.39	6.64	2.53	1.22	2595.89

**Table 7:** The conversion matrix of land use types in Shaanxi Province from 2000 to 2010 (km^2^).

Land use type in 2000	Land use type in 2010						
	Woodland	Grassland	Cropland	Wetland	Water body	Barren land	Build-up land
**Woodland**	45213.97	565.76	467.04	5.05	8.84	29.83	64.53
**Grassland**	1264.58	73279.46	2833.38	19.49	58.86	114.80	160.46
**Cropland**	1480.41	4713.62	64518.05	56.50	114.41	20.18	1052.79
**Wetland**	4.48	29.20	109.94	469.85	126.74	0.91	6.64
**Water body**	7.14	56.72	76.22	159.13	719.66	1.11	5.26
**Barren land**	14.00	239.13	80.40	3.16	12.70	4344.00	59.44
**Build-up land**	24.04	19.84	167.16	1.73	6.41	0.09	2930.63

**Table 4** shows that during the 1980s to 2010, most of the lost Cropland was converted into Grassland (4639.24 km^2^), Woodland (1550.64 km^2^), and Build-up land (1379.18 km^2^). This variation resulted from urbanization process and the GfG project after 2000. According to **Table 3**, Woodland and Grassland areas increased from 2000 to 2010, and the conversion matrix from 2000 to 2010 (**Table 7)** shows that the increase in Woodland and Grassland has resulted from Cropland reduction. This finding confirmed the effects of the GfG project. It is also consistent with the results of the study of You et al. 938) and Li et al. [39] in the Northern Shaanxi Province. These conversion matrices (**Tables 4–7**) have shown that the Build-up area has continued to increase, and the largest proportion of the increased Build-up area was transformed from Croplands in all the periods. Maps in **S1 Fig** showed where the changes have occurred from 1980s to 1995, from 1995 to 2000, from 2000 to 2010, and from 1980s to 2010.

### Changes in the ecosystem services value

**Fig 3:**
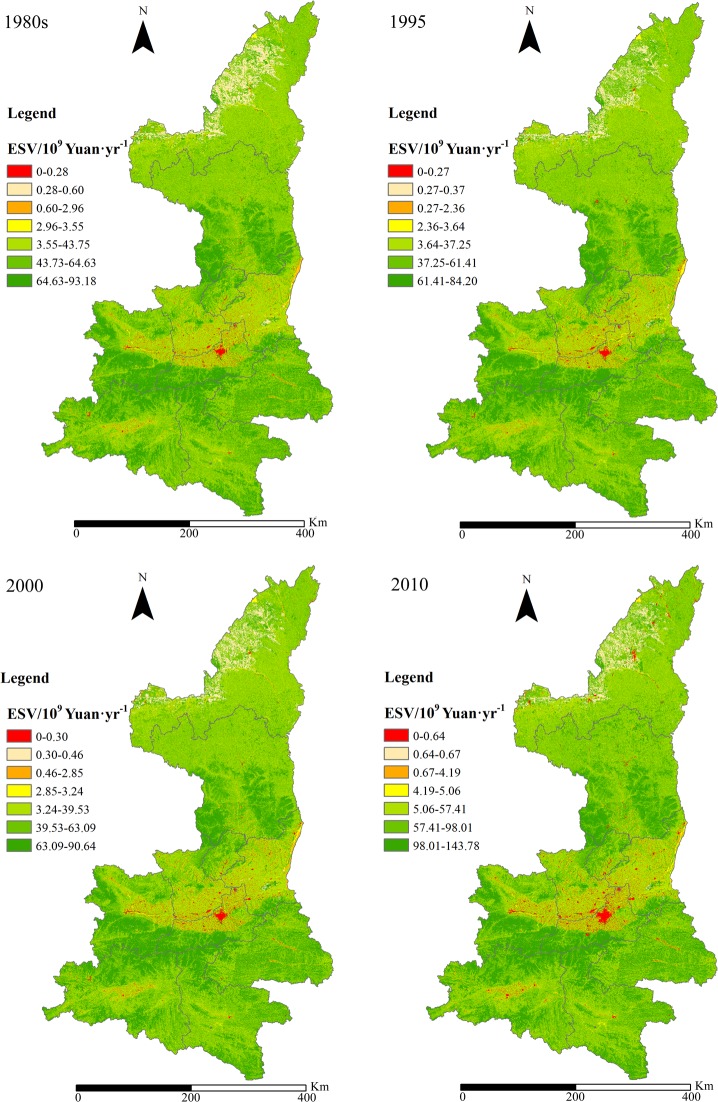
ESV distribution maps of Shaanxi Province in the 1980s, 1995, 2000 and 2010.

**Fig 4:**
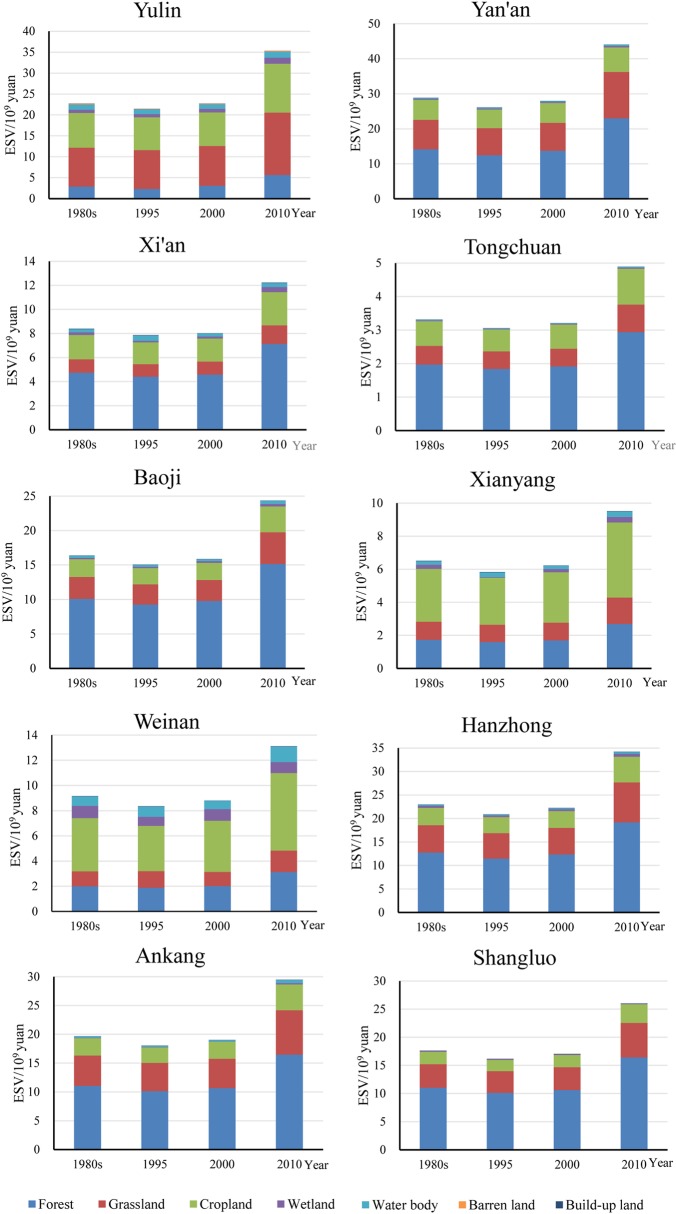
Value of ecosystem services in each city of Shaanxi Province from the 1980s to 2010.

Based on **Table 2**, ESV for the four years for the 10 cities were calculated by **Eqs (1–3)** and the results are shown in **Table 8.** The spatial distribution total ESVs of the province for the four years can be found in **Fig 3.** The ecosystem services values of the 10 prefecture cities for the 1980s and the years of 1995, 2000, and 2010 were obtained as shown in **Fig 4**.

**Table 8:** ESVs in Shaanxi Province from the 1980s to 2010 (10^8^ Yuan·yr^-1^).

	Woodland	Grassland	Cropland	Wetland	Water body	Barren land	Build-up land	Total
**1980s**								
**Gas regulation**	143.15	83.07	39.87	1.30	0.40	0.26	0.12	268.17
**Climate regulation**	134.87	86.39	53.72	7.32	1.61	0.56	0.26	284.73
**Water supply**	135.53	84.18	42.64	7.26	14.70	0.30	0.14	284.75
**Soil formation and protection**	133.21	124.05	81.41	1.07	0.32	0.74	0.34	341.14
**Waste treatment**	56.99	73.10	76.98	7.78	11.63	1.13	0.52	228.12
**Biodiversity protection**	149.45	103.56	56.49	1.99	2.69	1.73	0.79	316.70
**Food**	10.94	23.81	55.38	0.19	0.41	0.09	0.04	90.86
**Raw material**	98.75	19.94	21.60	0.13	0.27	0.17	0.08	140.94
**Recreation and culture**	68.92	48.18	9.41	2.53	3.48	1.04	0.48	134.04
**Sum**	931.80	646.28	437.50	29.58	35.51	6.02	2.76	2089.46
**1995**								
**Gas regulation**	129.36	78.93	33.95	1.04	0.41	0.16	0.12	243.97
**Climate regulation**	121.87	82.09	45.73	5.84	1.65	0.35	0.25	257.80
**Water supply**	122.47	79.98	36.31	5.79	15.07	0.19	0.14	259.95
**Soil formation and protection**	120.38	117.87	69.31	0.86	0.33	0.45	0.33	309.53
**Waste treatment**	51.50	69.46	65.54	6.21	11.93	0.70	0.51	205.84
**Biodiversity protection**	135.05	98.40	48.09	1.59	2.75	1.07	0.78	287.74
**Food**	9.88	22.63	47.15	0.16	0.43	0.05	0.04	80.33
**Raw material**	89.23	18.94	18.39	0.10	0.28	0.11	0.08	127.14
**Recreation and culture**	62.28	45.78	8.02	2.02	3.57	0.64	0.47	122.78
**Sum**	842.03	614.09	372.48	23.61	36.42	3.72	2.70	1895.06
**2000**								
**Gas regulation**	139.25	81.10	36.03	1.26	0.36	0.20	0.13	258.32
**Climate regulation**	131.19	84.34	48.54	7.06	1.47	0.43	0.28	273.31
**Water supply**	131.83	82.18	38.53	7.00	13.43	0.23	0.15	273.35
**Soil formation and protection**	129.58	121.11	73.56	1.04	0.29	0.56	0.37	326.50
**Waste treatment**	55.44	71.37	69.55	7.50	10.62	0.86	0.57	215.91
**Biodiversity protection**	145.37	101.10	51.04	1.92	2.45	1.32	0.88	304.09
**Food**	10.64	23.25	50.04	0.19	0.38	0.07	0.04	84.60
**Raw material**	96.06	19.46	19.52	0.12	0.25	0.13	0.09	135.63
**Recreation and culture**	67.05	47.04	8.51	2.44	3.18	0.79	0.53	129.53
**Sum**	906.40	630.94	395.31	28.52	32.44	4.59	3.04	2001.24
**2010**								
**Gas regulation**	220.88	125.98	52.32	1.84	0.57	0.29	0.27	402.16
**Climate regulation**	208.10	131.02	70.49	10.37	2.30	0.62	0.59	423.50
**Water supply**	209.12	127.66	55.95	10.29	20.94	0.34	0.32	424.62
**Soil formation and protection**	205.54	188.13	106.82	1.52	0.46	0.82	0.78	504.07
**Waste treatment**	87.94	110.86	101.01	11.02	16.57	1.25	1.19	329.84
**Biodiversity protection**	230.60	157.06	74.12	2.82	3.83	1.92	1.83	472.18
**Food**	16.87	36.12	72.67	0.28	0.59	0.10	0.09	126.71
**Raw material**	152.37	30.24	28.34	0.18	0.39	0.19	0.18	211.89
**Recreation and culture**	106.35	73.07	12.35	3.59	4.95	1.15	1.10	202.57
**Sum**	1437.78	980.15	574.08	41.93	50.59	6.68	6.36	3097.56

The total ecosystem service values of Shanxi Province were approximately 208.95 billion Yuan in the 1980s, 189.51 billion Yuan in 1995, 200.12 billion Yuan in 2000, and 309.76 billion Yuan in 2010. These values indicate that during the 1980s-2010, there was a brief decline with a turning point in 1995, which led to a sustained increase in the ESV. During the 1980s-2010, the largest increase was observed in Woodland and the Grassland ecosystem service values with an increase of 50.60 billion Yuan·yr^−1^ and 33.39 billion Yuan·yr^−1^, respectively. This increasing trend mainly ascribed to the fact that values of ecosystem services per unit area of Woodland and Grassland are higher than those of the other ecosystems. Meanwhile, it was also benefited from the GfG project and the execution of ecological restoration measures in Shaanxi Province to increase the area of Woodland and Grassland ecosystems. From the perspective of the ecosystem’s contribution to the value, the Woodland, Grassland and Cropland land use types showed the largest contribution to the ESV, accounting for more than 44%, 31%, and 19% of the ESV total, respectively, for all the four years due to their larger area of coverage and high unit ESVs. From the perspective of ecosystem services, soil formation and retention, which are provided by Woodland and Grassland ecosystems, were always at the top of the list. Generally, the values of the other services, in descending order, were waste treatment, biodiversity protection, water supply, climate regulation, gas regulation, raw material, food, and recreation and culture.

From **Table 8** and **Fig 4**, during the 1980s to 2010, the ESVs of nearly all the 10 cities have kept increasing since 1995, following a decrease from the 1980s to 1995. In 2010, the ESVs of the 10 cities presented the largest values in the study period. As for the specific cities, the dominating contributor of land use types varies. Grassland contributed most to ESV in Yulin city, Cropland is the greatest contributor in Weinan and Xianyang, and Woodland contribute the largest portion in the other seven cities.

### Regional disparities of the GDP and ESV

According to statistical data [44,45], the GDP values (in 2010 price) for 1985, 1995, 2000, and 2010 were 52.32, 165.59, 209.94, and 1007.04 billion Yuan, respectively, indicating an accelerating economic development. This variation is different from the variation of the ESVs described above.

The disparity of economic development denoted by GDP and eco-environmental conditions represented by ESV can be quantitatively analyzed using **Eqs (4) and (5)**, and the results were shown in **Table 9.** All the values of *Cv* of GDP are greater than those of ESV, this indicated that disparity of GDP is bigger than that of ESV. The values of *Cv* showed that the regional disparity in GDP experienced increasing process from 0.8079 in the 1980s to 0.9494 in 2000, and then decreased to 0.8613 in 2010, while ESV spatial disparity showed little changes from the 1980s (0.5277) to 2010 (0.5446). As for the GDP per capita, *Cv* in 1980s is 0.3936, and kept stable till 1995, then increased apparently in 2000 and 2010, indicating that the economic developing level is quite different in the ten cities from the 1980s to 2010, and the disparity was enlarged from 2000 to 2010. On the contrary, values of *Cv* of ESV per capita showed a decreasing trend from the 1980s to 2010 although they are all greater than values of *Cv* of GDP per capita. For all the four years, values of *Cv* in descending order are GDP, ESV per capita, ESV, and GDP per capita.

**Table 9:** The Variation Coefficient and Theil index of the GDP and ESV in Shaanxi Province.

Index	Item	1980s	1995	2000	2010
***Cv***	**GDP**	0.8079	0.8497	0.9494	0.8613
	**ESV**	0.5277	0.5269	0.5330	0.5446
	**GDP per capita**	0.3936	0.3941	0.4622	0.5092
	**ESV per capita**	0.7391	0.7040	0.7112	0.6860
**T**	**GDP**	0.6252	0.6451	0.6707	0.5005
	**ESV**	0.0181	0.0163	0.0161	0.0158

The values of the Theil index for the GDP show that the disparity in the GDP exhibited an increasing trend from 0.6252 in the 1980s to 0.6451 in 1995 and 0.6707 in 2000, and the situation improved in 2010 with a value of 0.5005, this variation trend is consistent with the findings of *Cv* of GDP. In contrast, the values of the Theil index for the ESV were very low which indicates that the regional disparity per area in the ESVs was small. A comparison of the values of the Theil index of the GDP and ESV showed that the values of GDP and GDP per capita were much larger than that of ESV, indicating that the regional disparity in economic development is still much larger than that of eco-environmental conditions even if the areas of cities are considered.

**Fig 5:**
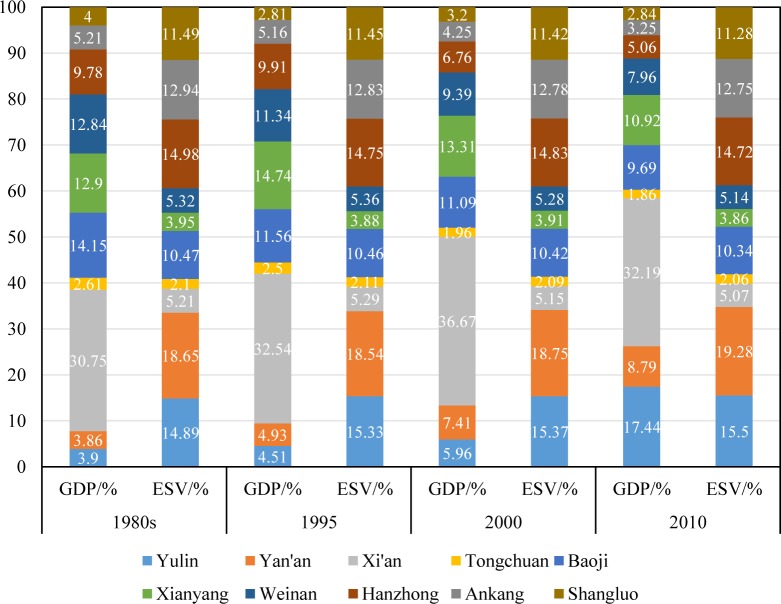
The contribution percentages of the GDP and ESV of each city to the provincial total.

Detailed exploration of the differences in regional disparities of the GDP and ESV can provide decision makers with helpful information. The percentages of the GDP and ESV of each city in the provincial total are shown in **Fig 5.**

In the 1980s, Yan’an, Hanzhong, Yulin, and Shangluo are the four top contributors to the provincial ESV total, while their contribution in GDP are quite small. On the contrary, Xi’an, Xianyang, Weinan, Baoji and Tongchuan contributed much more to GDP than to ESV. Particularly, Xi’an, the provincial capital, contributed nearly 6 times greater to GDP than to ESV, while Yan’an contributed nearly 5 times greater to ESV than to GDP. In 1995, the cities contributing to provincial ESV total in descending order, are Yan’an, Yulin, Hanzhong, Ankang, Shangluo, Baoji, Weinan, Xi’an, Xianyang and Tonchuang, while the order changed to Xi’an, Xianyang, Baoji, Weinan, Hanzhong, Ankang, Yan’an, Yulin, Shangluo and Tongchuan in term of GDP contribution. In this year, Shangluo, Yan’an and Yulin contributed 4.08, 3.76 and 3.4 times greater to ESV than to GDP, while Xi’an and Xianyang contributed 32.54% and 14.75% to the total GDP and their contributions to the total ESV were only 5.29% and 3.88% respectively. In 2000, Xi’an contributed nearly 7 times greater to GDP than to ESV. Shangluo, Ankang, Yulin, and Yan’an contributed 3.57, 3.02, 2.58 and 2.53 times greater to ESV than to GDP. The other cities showed similar characteristics of contribution to GDP and ESV as they did in 1995. In 2010, the sharing of Xi’an to GDP decreased although it still acted as the greatest contributor. The contributions of Yulin, Yan’an to GDP increased apparently while their contribution to ESV did not changed much. Shangluo, Ankang and Hanzhong contributed 3.97, 3.93 and 2.91 time greater to ESV than to GDP. In general, the cities with large contribution to GDP usually contributed little to ESV, and vice versa. Yulin and Yan’an developed rapidly due to oil and gas exploitation, their sharing in GDP increased 2.27 and 4.77 times from the 1980s to 2010, and their contribution to provincial total ESV were large as well owning to their large areal coverage to some extent. Xi’an contributed more than 30% to GDP but about 5% to ESV during the study period.

**Fig 6:**
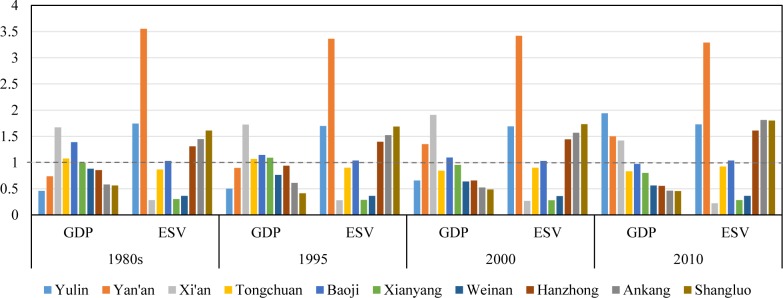
The ratios of GDP and ESV per capita of the ten cities to the provincial average values.

If the population of each city is considered in comparing GDP and ESV, more information can be provided for decision makers to seek choices for equitable development. The GDP per capita of the whole province were 1742.98 Yuan, 4824.97 Yuan, 5855.35 Yuan and 26960.66 Yuan in the 1980s, 1995, 2000 and 2010, respectively, and ESV per capita were 6960.81 Yuan, 5521.83 Yuan, 5581.57 Yuan and 8292.85 Yuan in the same temporal order. **Fig 6** showed the ratios of GDP or ESV per capita of the ten cities to the provincial average.

In 1980s, the GDP per capita of Xi’an and Tongchuan were greater than provincial average while their ESV per capita were both below the provincial average. The ESVs per capita of Yulin, Yan’an, Hanzhong, Ankang and Shangluo were greater than provincial average while theirs GDP per capita were all below the provincial average. Baoji was the only city that both GDP and ESV per capita are above provincial levels. As for the other four cities, both GDP and ESV per capita were below provincial averages. The situation did not change much in 1995 except that GDP per capita in Xianyang increased and surpassed provincial average. In 2000, the ESV per capita in the ten cities did not change in their order although the ratios of the cities changed a little. The GDP per capita in Tongchuan and Xianyang, two traditional industrial cities, decreased to below the provincial average, while Yan’an experienced economic increasing with GDP per capita above the provincial average. In 2010, the GDP per capita of Xi’an was greater than provincial average while its ESV per capita was the smallest among all the cities, far below the provincial average. The GDP per capita of Yulin and Yan’an were both greater than that of Xi’an, and their ESV per capita were all greater than provincial average as well, this is consistent with the rapid economic development, large areal coverage and eco-environmental protection since the late 1990s. The ESV per capita of Shangluo, Ankang, and Hanzhong were all above provincial average while their GDP per capita were still below the provincial average as they encountered in the 1980s, 1995 and 2000. Situations in the other cities were not optimistic, both GDP and ESV per capita in these cities were all below the provincial averages, which imply that they were confronted with poor and unfavorable eco-environmental conditions at the same time.

**Figs 5** and **6** showed that imbalance in both economy and eco-environmental conditions kept worsening from the 1980s to 2010, although all cities experienced increase in GDP and ESV, both in prefectural total and per capita. This implies the goal of regional equality was not achieved in economic development and ecological protection.

## Discussion

The study mainly adopted the aggregate benefit transfer method proposed by Costanza et al. [1] and Xie et al. [15, 16] to calculate ecosystem value over large spatial and temporal scales. In this method, values for the ecosystem services were estimated by values of unit area multiplying with the area of each ecosystem. The method itself has some shortcomings, such as, it is difficult to estimate the values of unit area of ecological services, and the spatial heterogeneity of ecosystem services value is not considered [16]. Another error source of the present study lies in matching ecosystem/biome classes with the land cover categories [16]. The ecosystems/biomes used as proxies for the land cover categories are clearly not perfect matches in every case [20]. Owning to the complexity and uncertainty [49–51], accurate calculation of absolute value of ecosystem services is not easy and always disputable. Besides the method used in this study, there are some other methods for estimating ESV such as those used in Chen and Zhang [52], Daily and Matson [49], Yu and Bi [53], and recently Ouyang et al. [54]. Using the invest-based method, Ouyang Z et al. [54] estimated the ecological services in China and concluded that ecological services increase in Loess Plateau (whose area mainly overlaps the northern part of Shaanxi Province) in the western China from 2000 to 2010. His result is consistent with the result obtained in the present study although quantitative comparison cannot be made due to lack of data.

Which method for ecological service valuation is more reliable is still on debating [53]. Different methods may lead to different estimation values, which will cause scientific critics and doubts on ecosystem service valuation. It is important to realize that accurate coefficients are often less critical for time series than cross-sectional analysis because coefficients tend to affect estimates of directional change less than estimates of the magnitude of ecosystem values at specific points in time [14,20,21]. In this study, the time series ESV were estimated, and spatial distribution of ESV are compared between 10 cities, thus, uncertainties and errors could be reduced or offset. It is predictable that the characteristics of ESV variation over time and spatial disparity revealed in the present study should not change much when other ESV valuation methods are adopted. In addition, most previous studies on ESV valuation in China [17–25] used the method based on [1, 15, 16], the ESV results in the present study, therefore, can be directly compared with those studies to find regional variations of ESV.

The present study revealed that the variances in the GDP and ESV have different characteristics among the ten cities. It implied unbalanced development between cities in the province. The economically backward cities took heavy on the supply of ecological service value. In China, ecological compensation is generally considered as an institutional arrangement regulating economic interests among ecological protectors, beneficiaries and destructors [55]. The provincial government took charge of the eco-compensation policy in China [56]. The ecosystem services value distribution and regional disparities of GDP and ESV in Shaanxi province can provide a concise and understandable way for Shaanxi provincial government to consider introducing ecological compensation policy to pursue regional equality. In China, administrative system is a hierarchical one, if data in details are available, with the method used in the present study, the ESV and GDP spatial disparities at city or county level can also be estimated. The results will be conducive to take more accurate measures to achieve balanced development.

## Conclusions

The total ESV for Shaanxi Province decreased from the 1980s to 1995 and 2000, and then increase to 2010, reflecting the positive effect of the national eco-environmental improvement project—Grain for Green–on the recovery of regional ESV in general. The three types of land use that made the largest contributions to the ESV were Woodland, Grassland, and Cropland. Soil formation and retention, waste treatment, and biodiversity protection were the three highest ESVs in all years of the study period. The ESVs in the 10 cities showed variation trends similar to those of the entire province.

The regional disparities in the GDP and ESV reflected a growing imbalance in economic development and eco-environmental conditions between cities under the same national project. For all the four years, values of *Cv* in descending order are GDP, ESV per capita, ESV, and GDP per capita. The Theil indexes of GDP were much greater than those of ESV. Regional disparity in GDP per capita kept increasing from the 1980s to 2010. Generally, the cities with large contribution to GDP contributed little to ESV. However, the shrinking of traditional industrial cities greatly reshape the spatial relation between GDP and ESV in Shaanxi since 2000.

The aim to calculate ESV is to raise the attention on the value of natural ecosystem that can hardly be captured by market transactions. However, even with the monetary value that was represented by the ESV has been presented, we still need some mechanism to direct social resources to be devoted into the protection of the ecosystems that provide ESV. The top-down approach that was used in the national eco-environmental improvement project like Grain for Green could provide an effective mechanism to broadly restore the function of natural ecosystem. However, with the increasing regional disparity of economic development, alternative approaches from bottom up for inter-regional eco-compensation calls for detailed studies on the spatial and temporal relations between the economic development and dynamics of ESV.

## Supporting information

S1 Fig Spatial distribution of land use changed and unchanged cells. (DOC)

S1 Table Land use type reclassification scheme. (DOC)

## References

[1] Costanza R, dArge R, deGroot R, Farber S, Grasso M, Hannon B, et al The value of the world’s ecosystem Services and natural capital. Nature. 1997; 387, 253–260.

[2] Daily GC. Nature’s services: Societal Dependence on Natural Ecosystems. Washington: Island Press, 1997.

[3] Costanza R, Daly M, Folke C, Hawwken P, Holling CS, McMichael AJ, et al Managing our environmental portfolio. Bioscience. 2000; 50,149–155.

[4] Ehrlich PH, Mooney HA. Extinction, substitution and ecosystem services. Bioscience. 1983; 33, 248–254.

[5] Costanza R, de Groot R, Sutton P, van der Ploeg S, Anderson SJ, Kubiszewski I, et al Changes in the global value of ecosystem services. Global Environ. Chang. 2014; 26,152–158.

[6] Burkhard B, Fath BD, Müller F. Adapting the adaptive cycle: Hypotheses on the development of ecosystem properties and services. Ecol. Model. 2011; 222, 2878–2890.

[7] Kubiszewski I, Costanza R, Franco C, Lawn P, Talberth J, Jackson T, et al Beyond GDP: Measuring and achieving global genuine progress. Ecol. Econ. 2013; 93: 57–68.

[8] Costanza R, d’Arge R, de Groot R, Farber S, Hannon B, et al The value of the world’s ecosystem services and natural capital. Ecol. Econ. 1998; 25, 3–15.

[9] Loomis J, Kent P, Strange L, Fausch K, Covich A. Measuring the total economic value of restoring ecosystem services in an impaired river basin: results from a contingent valuation survey. Ecol. Econ. 2000; 33, 103–117.

[10] Gascoigne WR, Hoag D, Koontz L, Tangen BA, Shaffer TL, Gleasonc RA. Valuing ecosystem and economic services across land-use scenarios in the Prairie Pothole Region of the Dakotas, USA. Ecol. Econ. 2011; 70, 1715–1725.

[11] Camacho-Valdez V, Ruiz-Luna A, Ghermandi A, Nunes PALD. Valuation of ecosystem services provided by coastal wetlands in northwest Mexico. Ocean. Coast. Manage. 2013; 78, 1–11.

[12] Yoshida A, Chanhda H, Ye YM, Liang YR. Ecosystem service values and land use change in the opium poppy cultivation region in Northern Part of Lao PDR. Acta Ecologica Sinaca. 2010; 30, 56–61.

[13] Palomo I, Martín-López B, Zorrilla-Miras P, Amo DGD, Montes C. Deliberative mapping of ecosystem services within and around Doñana National Park (SW Spain) in relation to land use change. Reg. Environ. Chang. 2014; 14, 237–251.

[14] Kreute UP, Harris HG, Matlock MD, Lacey RE. Change in ecosystem service values in the San Antonio area, Texas. Ecol. Econ. 2001; 39, 333–346.

[15] Xie GD, Zhen L, Lu CX, Xiao Y, Li WH Applying Value Transfer Method for Eco-Service Valuation in China. J. Resour. Ecol. 2010; 01, 51–59.

[16] Xie GD, Zhen L, Lu CX, Xiao Y, Chen C. Expert Knowledge Based Valuation Method of Ecosystem Services in China. Journal of Natural Resources. 2008; 23, 911–919.

[17] Wang Y, Gao J, Wang J, Qiu J. Value assessment of ecosystem services in nature reserves in Ningxia, China: A response to ecological restoration. PLoS ONE. 2014; 9(2): e89174 10.1371/journal.pone.0089174 24586571 PMC3929645

[18] Li J, Ren ZY. Variations in ecosystem service value in response to land use changes in the Loess Plateau in Northern Shaanxi Province, China. Int. J. Environ. Res. 2011; 5,109–118.

[19] Liu Y, Li JC, Zhang H. An ecosystem service valuation of land use change in TaiYuan City, China. Ecol. Model. 2012; 225, 127–132.

[20] Zhang P, He L, Fan X, Huo P, Liu Y, Zhang T, et al Ecosystem service value assessment and contribution factor analysis of land use change in Miyun County, China. Sustainability. 2015; 7, 7333–7356.

[21] Li TH, Li WK, Qian ZH. Variations in ecosystem service value in response to land use changes in Shenzhen. Ecol. Econ. 2010; 69, 1427–1435.

[22] Wang JH, Tian JJ, Li XY, Ma YJ, Yi WJ. Evaluation of concordance between environment and economy in Qinghai Lake Watershed, Qinghai-Tibet Plateau. J. Geogr. Sci. 2011; 21, 949–960.

[23] Cai YB, Zhang H, Pan WB, Chen YH, Wang XR. Land use pattern, socio-economic development, and assessment of their impacts on ecosystem service value: study on natural wetlands distribution area (NWDA) in Fuzhou city, southeastern China. Environ. Monit. Assess. 2013; 185, 5111–5123. 10.1007/s10661-012-2929-x 23054291

[24] Li G, Fang C. Global mapping and estimation of ecosystem services values and gross domestic product: A spatially explicit integration of national ‘green GDP’ accounting. Ecol. Indic. 2014; 46, 293–314.

[25] Sawut M, Eziz M, Tiyip T. The effects of land-use change on ecosystem service value of desert oasis: a case study in Ugan-Kuqa River Delta Oasis, China. Can. J Soil Sci. 2013; 93, 99–108.

[26] Sutton PC, Costanza R. Global estimates of market and non-market values derived from nighttime satellite imagery, land cover, and ecosystem service valuation. Ecol. Econ. 2002; 41, 509–527.

[27] Boumans R, Costanza R, Farley J, Wilsona MA, Portelab R, Rotmans J, et al Modelling the dynamics of the integrated earth system and the value of global ecosystem services using the GUMBO model. Ecol. Econ. 2002; 41, 529–560.

[28] Martínez ML, Pérez-Maqueo O, Vázquez G, Castillo-Camposc G, García-Francoa J, Mehltreter K, et al Effects of land use change on biodiversity and ecosystem services in tropical montane cloud forests of Mexico. Forest Ecol. Manag. 2009; 258, 1856–1863.

[29] Klug H, Jenewein P. Spatial modelling of agrarian subsidy payments as an input for evaluating changes of ecosystem services. Ecol. Complex. 2010; 7, 368–377.

[30] Kandziora M, Burkhard B, Müller F. Mapping provisioning ecosystem services at the local scale using data of varying spatial and temporal resolution. Ecosystem Services. 2013; 4, 47–59.

[31] Bateman IJ, Harwood R, Mace GM, Watson RT, Abson DJ, Andrews B, et al Bringing Ecosystem Services into Economic Decision-Making: Land Use in the United Kingdom. Science. 2013; 341, 45–50. 10.1126/science.1234379 23828934

[32] Yu SX, Shang JC, Guo HC. Evaluation of Ecological services of Jinlin Province, Northeast China. Chinese Geogr. Sci. 2004; 14, 215–220.

[33] Jia XQ, Fu BJ, Feng XM, Hou GH, Liu Y, Wang XF. The tradeoff and synergy between ecosystem services in the Grain-for-Green areas in Northern Shaanxi, China. Ecol. Indic. 2014; 43,103–113.

[34] Bai JJ, Di LP, Bai JT. NDVI and Regional Climate Variation since the Implementation of Revegetation Program in Northern Shaanxi Province, China. IEEE J-Stars. 2014; 7, 4581–4588.

[35] Zhou DC, Zhao SQ, Liu SG, Zhang LX. Modeling the effects of the Sloping Land Conversion Program on terrestrial ecosystem carbon dynamics in the Loess Plateau: A case study with Ansai County, Shaanxi province, China. Ecol. Model. 2014; 288, 47–54.

[36] Yin R, Zhao M. Ecological restoration programs and payments for ecosystem services as integrated social–ecological processes. Ecol. Econ. 2012; 73, 56–65.

[37] Liu J, Diamond J. Revolutionizing China's environmental protection. Science. 2008; 319, 37–38. 10.1126/science.1150416 18174421

[38] You YL, Jabbar MT, Zhou JX. Study of Environmental Change Detection Using Remote Sensing and GIS Application: A Case Study of Northern Shaanxi Province, China. Pol. J. Environ. Stud. 2012; 21, 783–790.

[39] Li WJ, Lu CH. Aridity trend and response to vegetation restoration in the loess hilly region of northern Shaanxi Province. J. Geogr. Sci. 2015; 25, 289–300.

[40] Uchida E, Xu JT, Scott R. Grain for Green: Cost-Effectiveness and Sustainability of China’s Conservation Set-Aside Program. Land Econ. 2005; 81, 247–264.

[41] Zhou HJ, Rompaey AV, Wang JA. Detecting the impact of the ‘Grain for Green’ program on the mean annual vegetation cover in the Shaanxi Province, China using SPOT-VGT NDVI data. Land Use Policy. 2008; 26, 954–960.

[42] Liu JY, Kuang WH, Zhang ZX, Xu XL, Qin YW, Ning J, et al Spatiaotemporal characteristics, patterns and causes of land-use changes in China since the late 1980s. J. Geogr. Sci. 2014; 24(2):195–210.

[43] Liu JY, Zhang ZX, Xu XL, Kuang WH, Zhou WC, Zhang SW, et al Spatial patterns and driving forces of land use change in China during the early 21st century. J. Geogr. Sci. 2010; 20(4): 483–494.

[44] Shaanxi Provincial Bureau of Statistics. Shaanxi statistical yearbooks 2011. Beijing: China Statistics Press 2011.

[45] National Bureau of Statistics of the People’s Republic of China. China Statistical Yearbook 2011. Beijing: China Statistics Press 2011.

[46] Conceição P, Ferreira P. The Young person’s guide to the Theil Index: suggesting intuitive interpretations and exploring analytical applications. UTIP Working Paper. 2000; 14.

[47] Coulter PB. Measuring inequality: A methodological handbook. Westview Press 1989.

[48] Xiao JY, Shen YJ, Ge JF, Tateishi R, Tang CY, Liang YQ, et al Evaluating urban expansion and land use change in Shijiazhuang, China, by using GIS and remote sensing. Landsc. Urban. Plan. 2006; 75(1–2):69–80.

[49] Daily GC, Matson PA. Ecosystem services: From theory to implementation. Proceedings of the National Academy of Science, USA. 2008. 10.1073/pnas.0804960105 PMC2474530 18621697

[50] Carpenter SR, Mooney HA, Agard J, Capistrano D, DeFries RS, Díaz S, et al Science for managing ecosystem services: Beyond the millennium ecosystem assessment. Proceedings of the National Academy of Science, USA. 2009; 106(5), 1305–1312. 10.1073/pnas.0808772106 PMC2635788 19179280

[51] Zhang Z, Wu CF, Tan R. Application of ecosystem service value in land use change research: Bottlenecks and prospects. Chin. J. Appl. Ecol. 2013; 24, 556–562. 23705405

[52] Chen ZX, Zhang XS. Value of ecosystem services in China. Chin. Sci. Bull. 2000; 45, 870–876.

[53] Yu ZY, Bi H. The key problems and future direction of ecosystem services research. Energy Procedia, 2011; 5, 64–68.

[54] Ouyang Z, Zheng H, Xiao Y, Polasky S, Liu J, Xu W, et al (2016) Improvements in ecosystem services from investments in natural capital. Science. 2016; 352 (6292):1455–1459. 10.1126/science.aaf2295 27313045

[55] Xie GD, Cao SY, Lu CX, Zhang CS, Xiao Y. Current status and future trends for eco-compensation in China. J. Resour. Ecol. 2015; 6, 355–362.

[56] Liu CL, Liu WD. Study of provincial differences and influential factors of eco-compensation in China. J. Natur. Resour. 2013; 29, 1091–1104.

