# Supplementary figures and images for: Spatial disparity dynamics of ecosystem service values and GDP in Shaanxi Province, China in the last 30 years
Source: PLoS One. 2017 Mar 30;12(3):e0174562. doi: 10.1371/journal.pone.0174562 (PMC5373591; doi:10.1371/journal.pone.0174562)

**S1 Fig** Spatial distribution of land use changed and unchanged cells


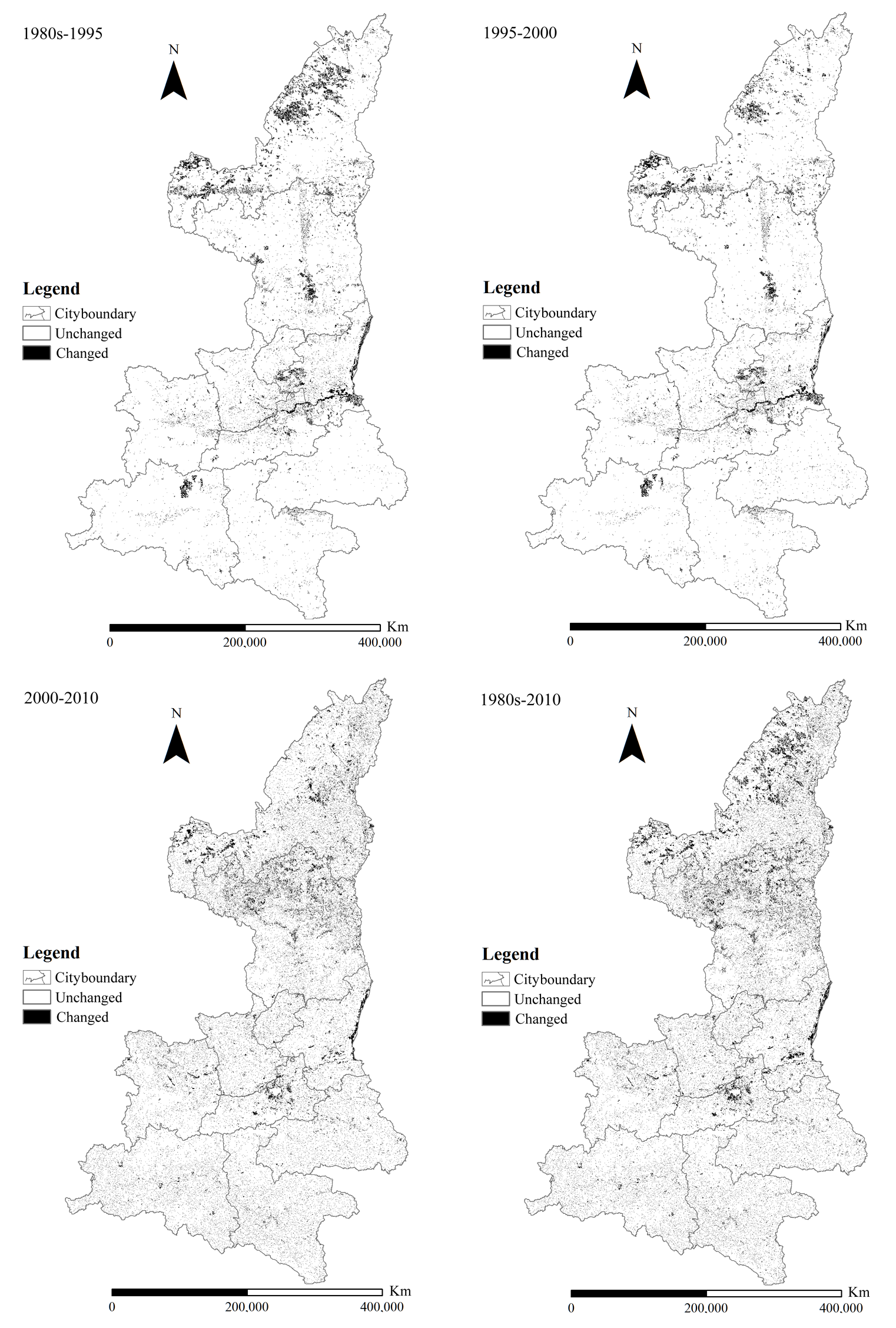

Supplement: S1 Fig — (DOC) [file pone.0174562.s001.doc]
