# Supplementary material for: Spatial disparity dynamics of ecosystem service values and GDP in Shaanxi Province, China in the last 30 years
Source: PLoS One. 2017 Mar 30;12(3):e0174562. doi: 10.1371/journal.pone.0174562 (PMC5373591; doi:10.1371/journal.pone.0174562)
Supplement: S1 Table — (DOC) [file pone.0174562.s002.doc]

**S1 Table. Land use type reclassification scheme**

| **Land use types in this study** | **Code** | **Land use classes** | **Descriptions** |
| --- | --- | --- | --- |
| Cropland | 11 | Paddy | Cultivated land of water supply and irrigation facilities, well irrigation in the general conditions, used to grow rice, lotus and other aquatic crops, including the rotation of paddy and upland crops cultivated land |
|  | 12 | Dryland | Cultivated land with no irrigation water source and facilities, rely on natural rainfall to grow crops; or with irrigation water source and facilities, in the general conditions, can normal irrigate upland crops. Cultivated land is given priority to with vegetables, fallow land and rotation plot |
| Woodland | 21 | Forest | Refers to the natural wood and plantation with over 30% canopy density. Including timber stands, economic forests and protective forests, etc. |
|  | 22 | Shrubbery | Refers to coppice-land and scrub woodland with over 40% canopy density, lower than 2 meters. |
|  | 23 | Opening forest land | Canopy density is 10%~30% |
|  | 24 | Other woodland | Immature forest land、slash、plant nursery and garden plot (orchard、mulberry field、tea garden, etc.) |
| Grassland | 31 | High coverage | Coverage > 50% of natural grassland, improved grassland and cutting grassland with good water condition and the thick grass growth |
|  | 32 | Middle coverage | Coverage was 20% ~ 50% of the natural grassland and improved grassland. This kind of grassland is general water deficiency and grass is relatively sparse |
|  | 33 | Low  coverage | Coverage was 5% ~ 20% of the natural grassland. This kind of grassland is water shortage and in sparse grass |
| Water body | 41 | Canal | Refers to natural or artificial excavation rivers and the land under perennial water level (artificial drainage including bank) |
|  | 42 | Lake | Refers to the natural formation of the ponding areas under perennial water level |
|  | 43 | Reservoir pits | Refers to man-made reservoir area land under perennial water level |
| Wetland | 45 | Tidal flat | Refers to the invasion zone between high tide and low water of coastal tide |
|  | 46 | Bottomland | Refers to the land between level period and flood period level of river/lake |
|  | 64 | Swamp | Refers to the land with flat low-lying terrain, poor drainage, wet for a long time, seasonal/regular waterlogged or water, surface with hygrophilous plant growth |
| Build-up land | 51 | Urban land | Refers to build-up area of the large, medium and small cities and county /town land |
|  | 52 | Rural residential  area | Refers to the rural residential areas |
|  | 53 | Other  build-up land | Refers to the factories and mines, large-scale industrial district, oil field, saltern, quarry and special land (traffic roads, airports) |
| Barren land | 61 | Sand land | Refers to the land of sand surface and vegetation coverage under 5%, including desert |
|  | 62 | Gobi | Refers to the land of surface is given priority to with crushed gravel, vegetation coverage under 5% |
|  | 63 | Saline land | Refers to the land of surface salinity accumulation, sparse vegetation, can only salt-tolerant plants growth |
|  | 65 | Bare land | Refers to the land of surface soil cover, vegetation coverage under 5% |
|  | 66 | Bare rock gravel land | Refers to the surface of rock or gravel, its land area covered under 5% |
